# Oral transmucosal fentanyl citrate analgesia in prehospital trauma care: a retrospective observational cohort study focusing on age and gender differences

**DOI:** 10.1186/s13049-026-01544-1

**Published:** 2026-01-06

**Authors:** Urs Pietsch, Anja Bommer, Björn Hossfeld, Volker Wenzel, Romano Meier, Roland Albrecht, Christoph Alexander Rüst

**Affiliations:** 1 Division of Perioperative Intensive Care Medicine, HOCH, Cantonal Hospital St. Gallen, Rorschacher Strasse 95, 9007 St. Gallen, Switzerland; 2 Swiss Air-Ambulance, Rega (Rettungsflugwacht/Guarde Aérienne), Zurich, Switzerland; 3 Department of Emergency Medicine, Inselspital Bern, University Hospital Bern, Bern, Switzerland; 4 Department of Internal Medicine, Cantonal Hospital Winterthur, Winterthur, Switzerland; 5 Department of Anesthesiology, Intensive Care Medicine, Emergency Medicine and Pain Medicine, and HEMS “Christoph 22” Ulm, Federal Armed Forces Hospital, Ulm, Germany; 6 Department of Anaesthesiology and Intensive Care Medicine, Friedrichshafen Regional Hospital, Friedrichshafen, Germany; 7 Department of Anesthesiology, University of Florida, Gainesville, FL USA; 8 Bergbahnen Lenzerheide Arosa, Lenzerheide, Switzerland; 9 Center for Interdisciplinary Emergency Medicine, Cantonal Hospital Winterthur, Winterthur, Switzerland; 10 EMS VGS Schweiz AG, Tübach, SG Switzerland; 11 EMS Regio 144 AG, Rüti, ZH Switzerland

**Keywords:** OTFC, Prehospital analgesia, Trauma support, HEMS

## Abstract

**Introduction:**

Effective pain management is essential in trauma care, as it reduces suffering and prevents long-term complications. However, prehospital analgesia is often inadequate, particularly in challenging environments such as alpine rescue settings, where establishing intravenous access can be difficult. Oral transmucosal fentanyl citrate (OTFC) offers a fast-acting, needle-free alternative, but has rarely been studied in civilian prehospital settings, particularly among paediatric and elderly populations. This study aimed to evaluate the feasibility, effectiveness and safety of OTFC for prehospital analgesia in a civilian mountain rescue context, covering all age and sex groups.

**Methods:**

This retrospective observational cohort study included all trauma patients treated with oral transmucosal fentanyl citrate (OTFC). Patients experiencing severe pain (NRS ≥ 4) and lacking intravenous access were eligible. OTFC was administered by trained emergency medical services providers. Data on demographics, pain scores and adverse events were prospectively recorded using a standardised digital protocol. The primary outcome was pain reduction, as measured by the Numeric Rating Scale (NRS); safety was assessed by recording adverse events. Statistical analysis included paired and unpaired tests, ANOVA and non-parametric equivalents, as appropriate. Significance was set at *p *< 0.05.

**Results:**

A total of 365 patients (60.5% male, mean age of 37 ± 18 years) were included in the study. The majority sustained extremity injuries, and the median initial pain score was 8 (IQR 6–8). Following OTFC administration, a significant median reduction of two NRS units (IQR1–3) was observed, corresponding to a mean relative reduction of 32% ± 22%. Pain reduction was consistent across all age and sex groups. In children and adolescents (6–16 years), the median reduction was 3 NRS points (relative reduction 34%), while in adults (> 16 years), it was 2 points (31% relative reduction). No significant differences in analgesic efficacy were found between subgroups based on age or sex. No serious adverse events, such as respiratory depression or the need for naloxone, were reported**.**

**Conclusion:**

OTFC is a safe, effective and practical analgesic for use in prehospital trauma care, especially in remote mountainous areas. Its consistent performance across age and sex groups, needle-free administration and low side-effect profile make it a valuable option where intravenous access is limited.

## Introduction

Treatment of pain caused by trauma is essential and reduces morbidity. Adequate analgesia reduces suffering and has a prophylactic effect on long-term outcomes such as chronic pain or post-traumatic stress disorder [1]. To achieve prompt analgesia, current pain treatment strategies aim at parenteral administration of drugs, since oral administration is slow and intramuscular administration is difficult to control.

The incidence of pain after trauma is reported to be up to 81%. Of this, 73% of pain is considered severe, 47% is rarely treated and 25% is inadequately treated. In tactical situations (e.g. military combat), only 15% of trauma patients receive analgesic treatment [2].

Ski slope accidents bring additional challenges such as extreme cold, wind, logistical constraints, evacuation and monitoring limitations, and more difficult intravenous access due to the hostile alpine environment, which may exacerbate inadequate analgesia in the prehospital setting.

Even during safe conditions, pain is only recognized in two-thirds of patients. Therefore, the National Association of EMS Physicians (NAEMSP) guidelines recommend that all patients be assessed for pain using the Numeric Rating Scale (NRS), administer opioid analgesics (morphine or fentanyl), repeat the pain assessment frequently, and re-administer analgesics if significant pain persists.

As intravenous administration is complex, time-consuming and can be disadvantageous in a hostile environment, the military (e.g. in the US, but also in the German Armed Forces) uses oral transmucosal fentanyl citrate (OTFC) lollipop. Oral transmucosal fentanyl citrate is a formulation that has emerged as a potential solution to this challenge, as it allows for rapid absorption through the oral mucosa and rapid onset of analgesia, potentially making it a valuable tool for emergency responders tasked with managing acute pain in the field [3–5]. The pharmacokinetic properties of oral transmucosal fentanyl offer several advantages over other routes of administration, such as the ability to bypass the gastrointestinal tract, which can result in higher bioavailability and more immediate pain relief for patients experiencing severe pain.

To date, there have been several studies of its efficacy and safety in the military environment, where its use has been established for years. There are some studies about its efficacy and safety in the civilian sector which have shown that it can be used efficiently and safely in adults. Little data is available on the gender-specific efficacy in paediatric or elderly patients. There is no data at all on the use in remote areas in this cohort.

The aim of the present study was to analyze whether the use of OTFC is feasibly in a mountain rescue setting in the Swiss Alps, where extrication requires an easy to use, fast-acting analgesic that can be safely administered by first responders and rescuers without the need for intravenous access.

In this observational cohort study, prehospital pain relief and adverse events were recorded. We hypothesized that the use of OTFC in trauma patients across all age and gender groups is feasible, effective and safe in remote and challenging environment.

## Methods

This retrospective observational cohort study included patients treated with OTFC between July 2020 and February 2024. The reporting of the study conforms to the STROBE statement for reporting of observational cohort studies. The local ethics committee reviewed and approved the study and waived written informed consent (BASEC No 2020–00.096 EKOS 21/006).

### Study design and participants

We included all patients with severe pain without IV access. Severe pain was defined as a score ≥ 4 on the numerical rating scale (NSR), where 0 is defined as absence of pain and 10 as the maximum imaginable pain. Administration of OTFC was contraindicated in case of altered consciousness, alcohol consumption, or apparent abnormal vital signs, which were defined as values of accepted clinical thresholds, including significantly elevated or decreased blood pressure, heart rate, or respiratory rate. An absolute contraindication for OTFC was head trauma (GCS < 15). There were other contraindications for the use of OTFC such as known allergy to the drug or its additives or patient refusal. Apnea and severe bradypnea with the need of ventilation and/or with the administration of naloxone were defined as major adverse events as an expression of overdosing.

All EMS providers, including ski patrol rescuers, EMS paramedics, and prehospital emergency physicians, received theoretical and practical instruction about OTFC. They were specifically instructed about the mechanism of action, indications and contraindications, as well as potential side effects. The study group consisted of trauma patients predominantly participating in recreational sport in the Ski and Bike resorts Lenzerheide, Arosa and St. Moritz (Switzerland), Ski World Cup (FIS Ski Worldcup St. Moritz, Lenzerheide Switzerland, Mountainbikde World cup (UCI World Cup Lenzerheide Downhill and Cross Country, Switzerland), Bike Park and MTB Stage Races (Swiss epic, Bike Giro Engadin, Switzerland).

### Primary and secondary outcomes

The primary outcome of the study was pain reduction, which was measured using the Numeric Rating Scale (NRS) both before and after OTFC administration. The secondary outcomes included the frequency and type of side effects, such as nausea, dizziness and respiratory depression. These outcomes were recorded in order to evaluate the effectiveness and safety of OTFC in prehospital trauma care.

### Drug

The medication used is OTFC 400 or 800mcg (Actiq, Teva Pharma AG, Basel, Switzerland).

Using this mode of drug administration, its onset is comparable to intravenous fentanyl, as it avoids hepatic first-pass effect. Intestinal absorption is minimal and can be considered negligible [6]. The EMS members participating in the study were additionally carrying a dose of 1.8mg naloxone (Nyxoid, Mundipharma Medical Company, Basel, Switzerland) for nasal application as an antidote. Administering additional medication occured primarily during HEMS deployment and only if it was indicated and a physician was on scene.

### Patient Enrollment and Group Stratification

Patients were retrospectively enrolled based on their medical records from the study period. The patients were stratified into two main cohorts: children and adolescents (6–16 years) and adults (≥ 17 years). Within each age group, patients were further subdivided based on the OTFC dosage administered. The dosing decision was made by the treating EMS provider based on an assessment of each patient's clinical condition, in particular their body weight (≥ 60 kg = 800 µg OTFC).

### Data collection and statistical analysis

The medical provider performed data collection, employing an app-based online protocol with predefined endpoints, prospectively recording the data in Table 1 (baseline characteristics). Using the protocol, we were able to analyze patient’s and mission’s characteristics, including sex, age, vital signs (heart rate, oxygen saturation, AVPU (Alert/Verbal/Pain/Unresponsive) scale, GCS and NRS) on initial presentation to the prehospital team. The incidence of adverse events, characterized as early cessation of OTFC administration, nausea/vomiting, drowsiness or respiratory depression determined the overall safety profile for OTFC. To ensure patient de-identification, no additional personal data were collected. Additionally, no personal data of the first responders on scene were collected, to make sure no conclusions about them could be drawn during later analysis.

**Table 1:** Baseline characteristics of patients treated with oral transmucosal fentanyl citrate (OTFC)

Variable	Total *n* = 365
*Age in years*	
Mean, ± SD	37 ± 18
6–16, n(%)	52 (14.2)
17–59, n(%)	267 (73.2)
≥ 60, n(%)	46 (12.6)
Male gender, n(%)	221 (60.5)
*Pain (NRS)*	
Initial, median (IQR)	8 (6–8) 7.4
After OTFC	5 (4–6)
Mean pain reduction	2 (1–3)
*Dose of OTFC*	
400 µg, n(%)	56 (15.3)
800 µg, n(%)	309 (84.7)
*Location of Trauma*	
Upper extremity, n(%)	215 (58.9)
Lower extremity, n(%)	133 (36.4)
Thorax, n(%)	15 (4.1)
Abdomen, n(%)	2 (0.5)
Spinal column, n(%)	18 (4.9)
*Adverse events*	
None, n(%)	323 (88.5)
Nausea/Vomiting, n(%)	25 (6.8)
Vertigo, n(%)	20 (5.5)
Dyspnea, n(%)	4 (1.1)
Major adverse events, n(%)	0 (0)
*Transport mode to hospital*	
Ground EMS, n(%)	280 (76.7)
Helicopter EMS, n(%)	85 (23.3)

#### Statistical analysis

Patients characteristics are summarized and presented in tables. Continuous variables were summarized by mean ± standard deviation if normally distributed, or by median and the interquartile range if skewed. To increase the quality of data analyses, each set of data was tested for normal distribution (D’Agostino and Pearson omnibus normality test) and for homogeneity of variances (F test) before statistical analyses. To find differences between paired groups (e.g. NRS before and after application of Fentanyl), a paired t-test was used in case of normal distributed data and a Wilcoxon matched-pairs signed rank test was used in case of not normal distributed data. To find differences between two unpaired groups, a Student’s t-test was used in case of normal distributed data (with Welch’s correction in case of unequal variances) and a Mann–Whitney test was used in case of not normal distributed data. To find differences between multiple unpaired groups (e.g. reduction in NRS after application of Fentanyl in different age groups), a one-way ANOVA and subsequent Tukey–Kramer multiple comparisons post-hoc test with a single pooled variance and multiplicity adjusted *P* value for each comparison was used in case of homoscedastic data with normal distributed residuals. In case of not normally distributed residuals, a Kruskal–Wallis test with subsequent Dunn’s multiple comparisons test was applied. Statistical analyses were performed using IBM SPSS Statistics (Version 24.0.0.0, IBM SPSS, Chicago, IL, USA) and GraphPad Prism (version 10.4.1) for Windows, GraphPad Software, San Diego, Ca, USA). Significance was accepted at *P* < 0.05 (two-sided for t-tests and exact for Mann–Whitney-tests).

## Results

A total of 365 patients (221 male sex, 60.5%; age, 37 ± 18 years) were treated with OTFC (Table 1) during the observation period. The upper extremity was the most frequently affected body region in 215 patients (58.9%), followed by injuries of the lower extremities in 133 cases (36.4%). The remaining 35 cases (9.5%) involved injuries to the thorax, spinal column, and abdomen. Some trauma victims suffered multiple injuries. A median pain level of 8 (IQR 6–8) on the numeric rating scale was documented during initial assessment by an EMS provider. Across the main subgroups, OTFC administration led to a statistically significant and clinically relevant median pain reduction of 2 NRS units (IQR 1–3), corresponding to a relative reduction of 32 ± 22%.

Before conducting a subgroup analysis, the patients were divided into two cohorts: children and adolescents (6–16 years) and adult (≥ 17 years). Among the younger patients, the median initial pain score was 8, decreasing to 5 after OTFC administration (IQR) 4–6). This corresponds to an absolute median pain reduction of 3 points (IQR 1–4) and a relative reduction of 34 ± 23%.

**Fig. 1:**
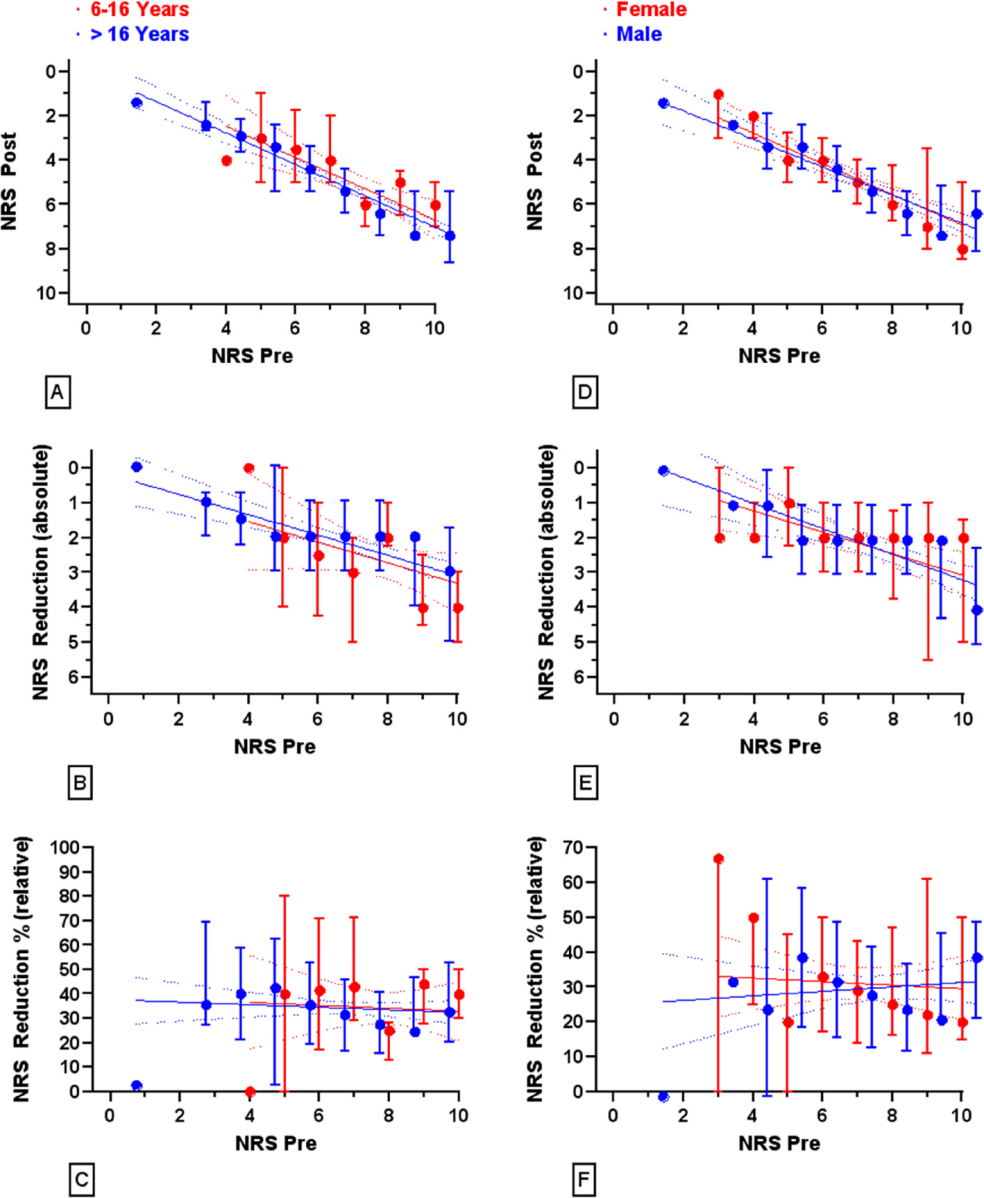
NRS after OTFC treatment (Panels **A** and **D**), the absolute NRS reduction (Panels B and E) and the percentage pain reduction (Panels **C** and **F**) depending on the initial NRS are shown for patients aged 6 to 16 years (red symbols and curves in Panels **A** to **C**) and over 16 years (blue symbols and curves in Panels **A** to **C**) as well as for all women (red symbols and curves in Panels **D**–**F**) and all men aged 17–59 years (blue symbols and curves in Panels **D**–**F**). All data are shown as median ± IQR

**Fig. 2:**
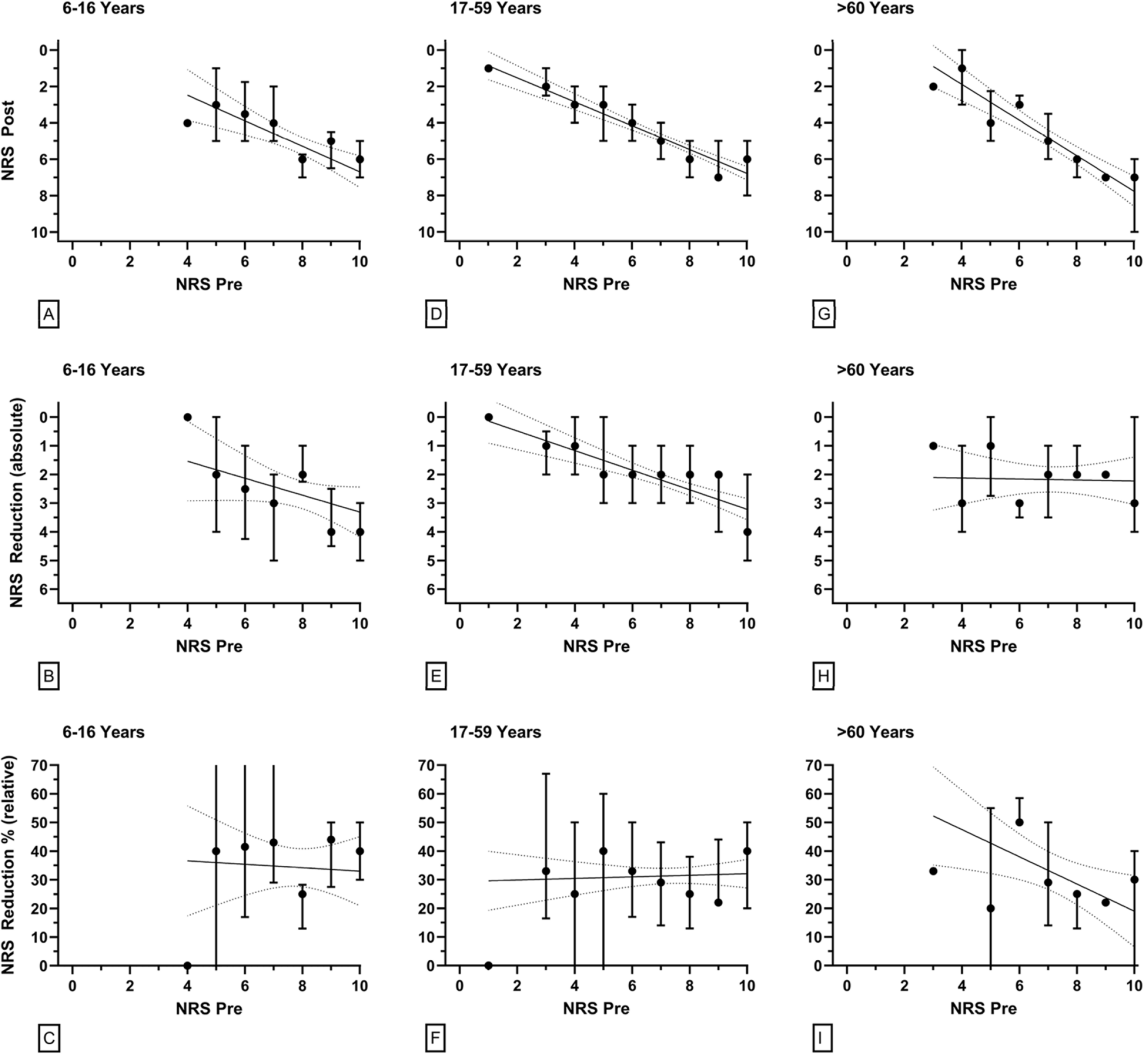
NRS after OTFC treatment (Panels **A**, **D**, **G**), the absolute NRS reduction (Panels **B**, **E**, **H**) and the percentage pain reduction (Panels **C**, **F**, **I**) depending on the initial NRS are shown for pooled female and male patients aged 6–16 years (Panels **A**-**C**), 17–59 years (Panels **D**-**F**) and > 60 years (Panels **G**-**I**), respectively. All data are shown as median ± IQR

Among adults, the median NRS decreased from 8 to 5 (IQR 4–6), with a median absolute reduction of two points (IQR 1–3) and a relative reduction of 31 ± 22%. (Figs. 1 and 2).

Inter-group analyses were performed according to age groups and gender to identify patterns in OTFC administration and outcomes. The results for the corresponding subgroups are summarised in Table 2.

**Table 2:** Pain reduction in different subgroups. The p-value refers to the within-group comparison of NRS before vs. after OTFC administration. A paired t test was applied

Subgroup	NRS initial,* n* (IQR)	NRS after OTFC,* n *(IQR)	NRS Reduction absolute, *n *(IQR)	Pain Reduction Percentage (SD)	*P* value
Male 6–16	8 (7–9)	5 (4.5–6.5)	2 (1–4)	32.9% (21.7%)	< 0.001
Female 6–16	8 (6–9)	5 (4–6)	3 (2–4)	37.8% (27.8%)	< 0.001
M&F 6–16	8 (7–9)	5 (4–6)	2.5 (1–4)	34.3% (23.5%)	< 0.001
Male 17–59 400 µg	8 (8–10)	6 (5–10)	2 (0–3)	21.0% (19.3%)	> 0.05
Female 17–59 400 µg	8 (8–10)	5 (4–8)	3 (0–5)	34.5% (27.6%)	< 0.001
M&F 17–59 400 µg	8 (8–10)	5 (4–8)	2.5 (0–4.25)	32.2% (26.4%)	< 0.001
Male 17–59 800 µg	8 (6–8)	5 (4–6)	2 (1–3)	31.4% (21.6%)	< 0.001
Female 17–59 800 µg	7 (6–8)	5 (3–6)	2 (1–3)	31.1% (21.8%)	< 0.001
M&F 17–59 800 µg	8 (6–8)	5 (4–6)	2 (1–3)	31.3% (21.6%)	< 0.001
Male ≥ 60 800 µg	7 (6–8)	5 (3–7)	3 (1–3.5)	33.7% (26.1%)	< 0.001
Female ≥ 60 800 µg	7 (5.25–8)	5.5 (3–7)	2 (1–3)	30.3% (21%)	< 0.001
M&F ≥ 60 800 µg	7 (6–8)	5 (3–7)	2 (1–3)	32.2% (23.8%)	< 0.001

Adult patients (age ≥ 17 years) were stratified into two age groups (17–59 years and ≥ 60 years), which were further subdivided by sex (female, male and pooled) and OTFC dosage (400 µg or 800 µg).

In the 17–59 age group, OTFC resulted in a significant median pain reduction across both dosage groups. For patients receiving 400 µg, the median reduction was 2.5 NRS points (IQR 0–4.25) male and females combined, corresponding to a 32.2% reduction (SD 26.4%). While female patients in this group showed a statistically significant pain reduction (*p* < 0.001), male patients did not reach statistical significance (*p* > 0.05). For the 800 µg group, both male and female patients exhibited a consistent and statistically significant median reduction of 2 NRS points (IQR 1–3), with pain reduction percentages ranging around 31% (SD ~ 22%), and all comparisons reaching statistical significance (*p* < 0.001).

In patients age ≥ 60, receiving the 800 µg dosage, a median pain reduction of 2 points (IQR 1–3) was demonstrated, representing a 32.2% (SD 23.8%) reduction in the combined group. Statistically significant reductions were achieved by both male and female subgroups (*p* < 0.001). The 400 µg group within this age category exhibited insufficient sample sizes to facilitate meaningful analysis.

In summary, no significant differences in the analgesic efficacy were observed between sexes or age categories within each dosage group.

For the paediatric population (children and adolescents aged ≤ 16 years), subgroup analyses revealed no statistically significant differences between younger children (6–13 years) and adolescents (14–16 years) in any parameter assessed. Consequently, the results are presented for the entire paediatric cohort as one group in order to facilitate clarity.

Amongst all paediatric patients, the median initial NRS was 8, decreasing to 5 after OTFC administration (IQR 4–6).

This finding corresponds to a median absolute reduction of 3 points (IQR 1–4) and a relative reduction of 34 ± 23%, indicating a clinically meaningful analgesic effect in this age group.

No sex-specific differences were observed in baseline pain or in absolute or relative NRS reduction within the paediatric population.

The detailed subgroup analysis originally performed showed comparable treatment effects without statistically significant variation, likely due to limitations in the sample size and wide variance in the smaller paediatric categories. Finally, we compared male and female patients aged 17–59 years who received an 800 µg dose of OTFC, stratified by their initial NRS scores. This analysis revealed no significant differences in baseline pain intensity or analgesic effect between the two groups, indicating that OTFC is equally effective in both men and women in this age group.

**Fig. 3:**
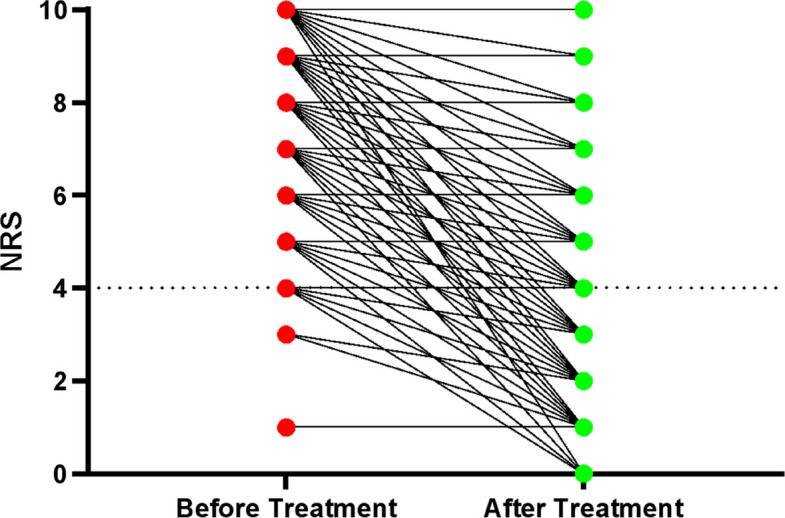
Initial NRS (left; red), NRS after OTFC treatment (right; green) and development of NRS (connection lines) during treatment of all pooled patients

The relative reduction in NRS scores was approximately 35% regardless of initial pain intensity. This suggests that OTFC might provide a consistently proportional analgesic effect, regardless of baseline pain severity. (Fig. 3).

Although children and adolescents appeared to experience a greater reduction in pain scores than adults, these differences were not statistically significant. Initial NRS scores were higher in children (mean 7.8 ± 1.4 for 6–16-year-olds) than in adults (7.3 ± 1.7 for 17–59-year-olds and 7.2 ± 1.8 for those aged 60 and over). Post-treatment NRS scores converged across all groups (5.2 ± 2, 5.0 ± 2.0 and 5.0 ± 2.3, respectively), suggesting comparable final pain levels. The absolute NRS reduction was slightly higher in the paediatric subgroups (2.7 ± 1.7) than in the adult subgroups (2.3 ± 1.7 and 2.2 ± 1.5), as was the relative reduction (34.3 ± 23.5% vs. 31.4 ± 21.9% and 32.3 ± 23.5%). However, none of these differences were statistically significant, likely due to wide variance and the small size of the pediatric sample.

A total of 279 patients (76.4%) had an NRS of 4 or higher after therapy.

Overall, the OTFC was well tolerated with only a few mild side effects reported, including nausea in 25 (6.8%) or vertigo in 20 (5.5%). A total of 4 cases (1.1%), were reported in which dyspnoea was identified as a side effect.

## Discussion

The main finding of this retrospective study is that OTFC is an effective treatment capable of significantly reducing pain in all age groups and in both men and women, with no significant side effects. Nevertheless, 76% of patients still had an NRS value above 4, a finding that has also been reported previously and appears independent of provider qualification [7, 8].

OTFC offers several advantages in the prehospital setting, including its rapid onset, repeatable titration, and no need for intravenous access. Unlike ketamine, OTFC does not induce psychotropic side effects, rendering it suitable for widespread use. These characteristics are of particular value in challenging prehospital environments.

Our data show that well-trained EMS providers and paramedics can administer OTFC safely and effectively, even with limited monitoring. Similar findings in PACKMaN confirm the hypothesis paramedics can deliver potent analgesia with a low incidence of serious adverse events [7]. OTFC therefore helps bridge prehospital care gaps when physician support is delayed.

A consistent and clinically meaningful pain reduction were observed across children, adolescents, adults and elderly patients, as well as both sexes. In patients aged 17–59 years, a median reduction of approximately 35% was achieved with 800 µg OTFC, regardless of baseline pain intensity. This highlights the reliability of OTFC across diverse demographic and operational settings.

In the context of paediatric patients, OTFC is a useful alternative when intravenous access is difficult and anxiety-inducing [9]. Although children presented with higher initial pain scores than adults, they achieved comparable post-treatment values, indicating a numerically stronger analgesic effect. While these differences did not reach statistical significance, likely due to the small sample size and variability within the paediatric cohort, the overall trend supports the clinical value of OTFC in younger trauma patients. Importantly, early and effective pain management might help reduce the risk of long-term complications, including chronic pain or post-traumatic stress disorder.

The present study also expands the evidence beyond the primarily military adult population described in earlier literature by showing consistent safety and efficacy in paediatric and geriatric civilians.

Its performance in alpine rescue further demonstrates its practicality. Cold, wind, geographic isolation and limited monitoring complicate IV placement, while OTFC’s needle-free route allows continuous analgesia during evacuation procedures.

Intranasal opioids represent another non-invasive option [10], however nasal congestion, secretions, or vasoconstriction in cold environments can impair absorption. OTFC avoids such limitations while enabling better dose control and a more established pharmacokinetic profile.

In comparison with other commonly used prehospital analgesics, OTFC fulfils several characteristics of an ideal agent: rapid onset, ease of use, minimal monitoring requirements and applicability across patient groups. Morphine requires IV access and close monitoring, while ketamine may induce dissociation if not titrated carefully. Methoxyflurane has fixed dosing and relevant contraindications.

Despite these advantages, suboptimal analgesia remains an issue, with 76% of patients exhibiting persistent NRS > 4. Similar findings were reported by Oberholzer et al. nd in PACKMaN, where neither ketamine nor morphine consistently achieved adequate analgesia [7, 8].

Inadequately treated pain can trigger harmful stress responses via hypothalamic–pituitary–adrenal activation,. Abnormal cortisol levels are associated with increased mortality [11, 12]. Consequently improving prehospital analgesia could therefore help mitigate neuroendocrine stress and might improve patient outcomes.

This study is limited by its retrospective design, precluding causal inference, and the reliance on EMS documentation, which likely underestimates adverse events. The absence of injury severity scoring and a comparator treatment group further restricts interpretation.

## Conclusions

Oral transmucosal fentanyl citrate (OTFC) is a safe, effective and feasible option for prehospital analgesia in a variety of patient groups and operational settings. It reliably reduces pain in children, adults, and elderly patients, even under challenging conditions or limited medical infrastructure. Its ease of use, rapid onset and non-invasive administration make it particularly valuable where intravenous access is difficult to achieve. However, despite these advantages, many patients still experience pain, highlighting the ongoing challenge of achieving adequate analgesia in prehospital care.

## Data Availability

The datasets used and analysed during the current study are available from the corresponding author on reasonable request.
